# Expanding frontiers in materials chemistry and physics with multiple anions

**DOI:** 10.1038/s41467-018-02838-4

**Published:** 2018-02-22

**Authors:** Hiroshi Kageyama, Katsuro Hayashi, Kazuhiko Maeda, J. Paul Attfield, Zenji Hiroi, James M. Rondinelli, Kenneth R. Poeppelmeier

**Affiliations:** 1 Graduate School of Engineering, Kyoto University, Nishikyo-ku, Kyoto 615-8581 Japan; 2 Department of Applied Chemistry, Kyushu University, Fukuoka, 819-0395 Japan; 3 Department of Chemistry, School of Science, Tokyo Institute of Technology, 2-12-1-NE-2 Ookayama, Meguro-ku, Tokyo 152-8550 Japan; 4 Centre for Science at Extreme Conditions, University of Edinburgh, EH9 3FD Edinburgh, UK; 5 Institute for Solid State Physics, University of Tokyo, Kashiwanoha 5-1-5, Kashiwa, Chiba 277-8581 Japan; 6 Department of Materials Science and Engineering, Northwestern University, Evanston, IL 60208 USA; 7 Department of Chemistry, Northwestern University, Evanston, IL 60208 USA

## Abstract

During the last century, inorganic oxide compounds laid foundations for materials synthesis, characterization, and technology translation by adding new functions into devices previously dominated by main-group element semiconductor compounds. Today, compounds with multiple anions beyond the single-oxide ion, such as oxyhalides and oxyhydrides, offer a new materials platform from which superior functionality may arise. Here we review the recent progress, status, and future prospects and challenges facing the development and deployment of mixed-anion compounds, focusing mainly on oxide-derived materials. We devote attention to the crucial roles that multiple anions play during synthesis, characterization, and in the physical properties of these materials. We discuss the opportunities enabled by recent advances in synthetic approaches for design of both local and overall structure, state-of-the-art characterization techniques to distinguish unique structural and chemical states, and chemical/physical properties emerging from the synergy of multiple anions for catalysis, energy conversion, and electronic materials.

## Abstract (web summary)

Inorganic compounds with multiple anions have added a new dimension to the discovery of materials. Here the authors review the recent progress in the development of mixed-anion compounds, focusing on the roles of multiple anions in synthesis, characterization and properties.

## Introduction

The continuing growth of many modern technologies is driven by the development of functional solid-state materials, such as metal oxides, fluorides, and nitrides that adopt a range of structural types and compositions. The accumulation of knowledge based on experimental data (or at times “chemical intuition”) and computational modeling and validations has led to extensive knowledge of these “single-anion” materials and affords further prediction of properties. Most of these results derive from variations in metal cation chemistry, as opposed to the anion, when examining structure-property relationships.

A multiple or mixed-anion compound is a solid-state material containing more than one anionic species in a single phase, such as oxyfluorides (oxide-fluoride) and oxynitrides (oxide-nitride). Unlike oxides, which exhibit diverse chemistries and structures often known from mineralogy, the structures of most mixed-anion compounds, among other aspects, are less explored with much to learn. This is readily seen when looking at the local structure of these compounds where the metal cation is bonded to more than one anionic ligand to form a heteroleptic polyhedron (Box 1). The different anionic characteristics, such as charge, ionic radii, electronegativity, and polarizability (Table 1) add new dimensions to control and tune the electronic and atomic structure of materials, which may support phenomena inaccessible to a single-anion analog.

**Table 1:** Basic parameters of anions-forming elements and their ions

	**Atomic properties**						**Anionic properties**		
	Isotope with non-zero nuclear spin, *I*^a^	Natural abundance (%)^b^	Neutron coherent scattering length (fm)^c^	Ionization energy (kJ/mol)^d^	Electron affinity (kJ/mol)^e^	Pauling’s electronegativity^f^	Formal valence/electronic configuration	Coordination number/ionic radius (pm)^g^	Polarizability (Å^3^)^h^
H			−3.7390	1312.0	72	2.20	–1	127–152	
	^1^H 1/2	99.985	−3.7406				[He]		
	^2^H 1	0.015	6.671						
N			9.36	1402.3	–8	3.04	–3	IV 146	
	^14^N 1	99.63	9.37				[Ne]		
O			5.803	1313.9	141	3.44	–2	II 135	1.68 (MgO)
	(^16^O)	99.762	5.803		(–780)		[Ne]	III 136	3.17 (BaO)
	^17^O 1	0.04	5.78					IV 138	1.79 × 10^−1.766/V^
	(^18^O)	0.2	5.84					VI 140	
								VIII 142	
F	^19^F 1/2	100	5.654	1681.0	328	3.98	–1	II 128.5	0.89 (LiF)
							[Ne]	III 130	1.36 (CsF)
								IV 131	0.82 × 10^−3.000/V^
								VI 133	
P	^31^P 1/2	100	5.13	1011.8	72	2.19	−3	212	
							[Ar]		
S			2.847	999.6	200	2.58	−2	VI 184	4.60 (MgS)
	(^32^S)	95.02	2.804		(–492)		[Ar]		6.41 (BaS)
	^33^S 3/2	0.76	4.74						
Cl			9.5770	1251.2	349	3.16	–1	VI 181	2.88 (LiCl)
	^35^Cl 3/2	75.77	11.65				[Ar]		3.47 (RbCl)
	^37^Cl 3/2	24.23	3.08						3.88 × 10^−1.800/V^
As	^75^As 3/2	100	6.58	947.0	78	2.18	–3	222	
							[Kr]		
Se			7.970	941.0	195	2.55	–2	VI 198	
	^77^Se 1/2	7.6	8.25				[Kr]		
Br			6.795	1139.9	325	2.96	–1	VI 196	3.99 (LiBr)
	^79^Br 3/2	50.69	6.80				[Kr]		4.67 (RbBr)
	^81^Br 3/2	49.31	6.79						
Sb			5.57	834	103	2.05	–3		
							[Xe]		
Te			5.80	869.3	190	2.10	–2	VI 221	
							[Xe]		
I	^127^I 5/2	100	5.28	1008.4	295	2.66	–1	VI 220	
							[Xe]		
Bi			8.532	703		2.02	–3		
							[Rn]		

^a^ Ref. ^105^; isotopes with zero nuclear spin are indicated in parentheses

^b^ Ref. ^105^

^c^ NIST center for neutron research, neutron scattering lengths and cross sections, https://www.ncnr.nist.gov/resources/n-lengths/

^d^ Ref. ^106^

^e^ Ref. ^106^; second electron affinity is indicated in parentheses

^f^ Ref. ^106^

^g^ Ionic radii with^107^ and without^106^ specifying the coordination number. Ionic radii for H are derived from those discussed in ref. ^108^

^h^ Values with chemical formula in parentheses are those experimentally estimated in compounds with rock salt structure^109^. The equations as a function of the anion molar volume, *V*, evaluated in ref. ^110^

Such anion-centered chemistry and physics is still in its infancy; there is much unexplored space, making it perhaps the most untapped field of materials sciences and giving new challenges and opportunities. In this review, we aim to describe the current status and scope, as well as outline future prospects and challenges surrounding mixed-anion (mostly oxide based) compounds, in particular, focusing on crucial roles of multiple anions in synthesis, characterization, and chemical and physical properties. Note that we had to be selective in materials and references because of the limited space. We provided mainly reviews or selected references, which could be an entry point to the literature search for readers who need additional information.

## Mixed-anion directed strategies

**Fig. 1:**
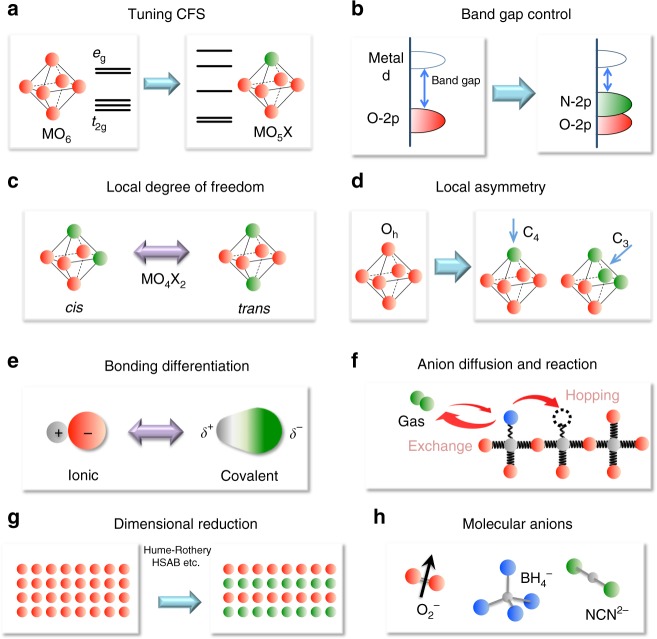
What mixed-anion compounds can do (Concepts 1a–1h). **a** Extensive tuning of CFS. Replacement of one oxygen with a different anion allows extensive tuning of CFS even when the octahedron stays rigid. **b** Non-oxide anion with lower electronegativity (vs. oxide) in semiconductors raises the VBM and narrows the band gap, affording visible light applications like water splitting catalysis^51,52^ and pigmentation^49^. **c** Local degree of freedom. An MO_4_X_2_ octahedron has *cis* and *trans* geometries, major parameters widely exploited in coordination chemistry, but less so in solid-state chemistry. When MO_4_X_2_ octahedra with *cis* or *trans* preference are connected to form an extended lattice, various non-trivial structures can appear, some of which have ‘correlated disorder’^34,38^. **d** Local coordination asymmetry. The O_h_ symmetry of the rigid octahedron is lost by replacing one and three ligands, leading to C_4_ and C_3_ symmetry. **e**, **f** Covalency and ionicity can be tuned to acquire desired functions. A weakly bonded ligand to a metal centre can generate functions related to anion diffusion (anionic conductivity) and anion reaction at the surface (catalysis), whereas the structural stability is secured by strongly bonded counter ligands^13,69,70^. **g** Dimensional reduction. Alternate stacking of layers of different anions, which can be rationalized utilizing, e.g., HSAB concept and Hume-Rothery rules^78^, have potential to enhance two-dimensionality, leading to novel properties, including high*-T*_c_ superconductivity^81,82,103^. **h** Inclusion of molecular anions further widens possibilities. Available parameters, include anisotropic shape, magnetic moment (e.g., *S* = 1/2 moment in O_2_^−^) and additional (anisotropic) bonding (e.g., bonding to hydrogen in BH_4_^–^)^99–101^

Understanding of mixed-anion compounds is still growing, but recent studies have unveiled several key features that are otherwise inaccessible in traditional single-anion compounds, as summarized in Fig. 1. Replacing oxide ligands in coordination octahedra or tetrahedra with other anions can differentiate the binding energy (Fig. 1e), which may benefit chemical reaction and anionic diffusion (Fig. 1f). It might also cause a (local) symmetry breaking (Fig. 1d) or create a *cis*/*trans* degree of freedom (Fig. 1c). The latter is a familiar ingredient in coordination chemistry, but less so in solid-state chemistry. Additionally, the crystal field splitting (CFS) can be tuned to the extent that is only allowed in coordination complexes, while retaining the original polyhedral shape and connectivity (Fig. 1a). An extensive modification of band (electronic) structures is also noteworthy, leading to a reduced dimensionality (Fig. 1g) and an upward shift of valence band maximum (VBM) (Fig. 1b).

**Fig. 2:**
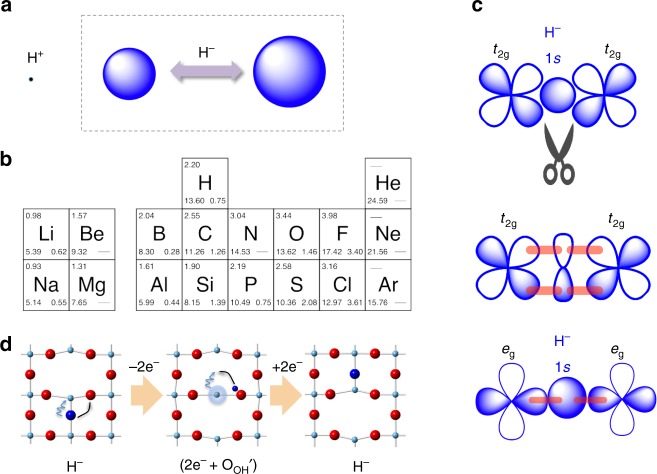
Specific features of hydride anion H^–^ (Concepts 2a–2d). **a** As opposed to other anions, H^–^ is highly flexible in size (right, exaggerated for clarity), with ionic radii of ∼130-153 pm found in metal hydrides. This means that H^–^ (or more precisely H^δ–^) can adapt itself to a given local environment. This appears to hold for oxyhydrides^12^ and is important for the hydride detection and characterization by ^1^H-NMR (Fig. 4b)^44^. A high-pressure study revealed that H^–^ is extremely compressible^9^. **b** A periodic table of elements, taken from ref. ^1^. Justifications of hydrogen positioning above carbon arise from a half filled outer shell and a similarity in electronegativity to group IV elements (C, Si…). **c** The lack of π symmetry in H^–^ 1*s* orbital allows this ligand to act as a “π-blocker” (or orbital scissors) with respect to *t*_2g_ orbitals of a transition metal, leading to the dimensional reduction in Fig. 1g^9,89^. A fairly strong σ bonding is suggested between *e*_g_ and H^–^ 1*s* orbitals^8^. **d** Hydride anion is regarded as a highly labile ligand, which, combined with the electron donating nature of hydride, allow versatile opportunities for oxyhydrides, including hydride anion conductivity^70^, topochemical reactions^13,14^, and catalysis^95^. Shown in this panel is a theoretically proposed non-trivial hydride diffusion process in SrTiO_3_^104^, involving electron transfer from/to the titanium cation, being analogous to the so-called proton coupled electron transfer (PCET)—“electron coupled hydride transfer” (ECHT). Fixation of such transient “two-electron released state” is realized in H^–^ ion-doped 12CaO·7Al_2_O_3_ by UV-light excitation^47^

Oxyhydrides (oxide hydrides), containing oxide and negatively charged hydride (H^–^) anions, are rare but can be remarkable materials. Several features specific to hydride are given in Fig. 2. Hydrogen is the simplest (and lightest) element with one electron and one proton, giving the hydride anion distinct characteristics that differentiate it from other anions. For example, its bipolar nature and moderate electronegativity allow covalent, metallic, and ionic bonding, depending on the electronegativity of the element with which hydrogen bonds. This is schematically represented by the unconventional periodic table of elements (Fig. 2b)^1^, where values of electronegativity, ionization potential, and electron affinity are shown in the upper left, lower left, and lower right corner of each box. Related to this, the extraordinary flexibility in size of hydride (Fig. 2a) and possible reactions involving the zwitterionic nature (Fig. 2d) may bring about unprecedented functions. The flexible nature of hydride is also evident in its polarizability, as the refractive index of LiH (1.985) is significantly larger than that of LiF (1.392) despite the fewer number of electrons. Finally, H^–^ is the only anion which does not possess *p* orbitals in the valence shell. The lack of *p* orbitals in the outermost shell (Fig. 2c) significantly distinguishes its chemical bonding nature and its magnetic interaction with other anions.

## Synthesis beyond heat and beat

**Fig. 3:**
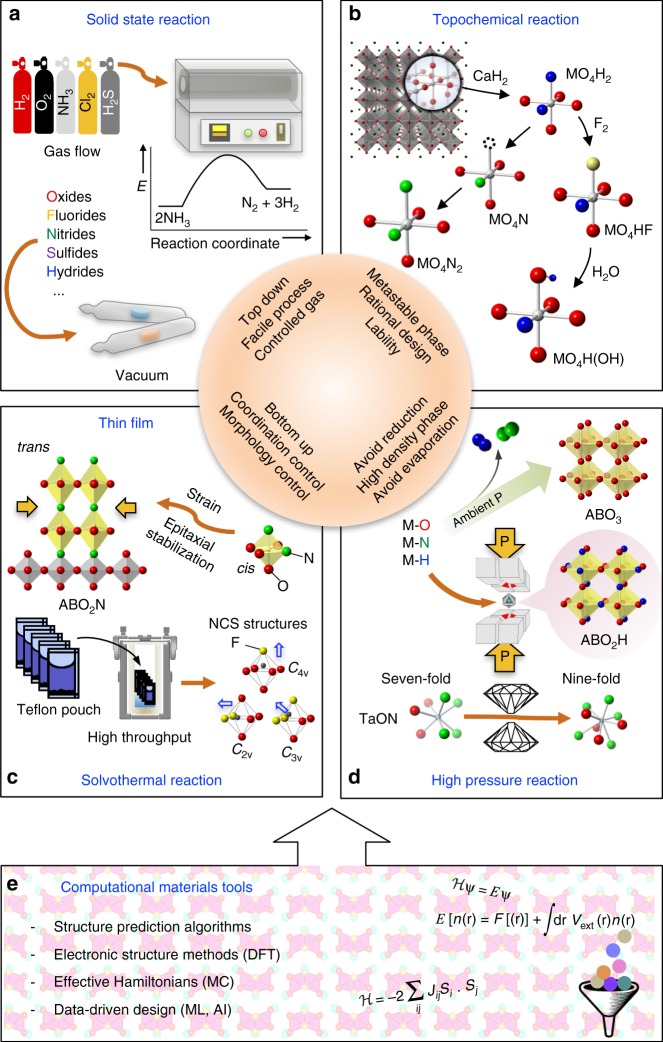
Synthetic approaches for mixed anion compounds. **a** Traditional high-temperature solid-state reactions. Controlled atmospheres, such as flowing gases (NH_3_, Cl_2_, CS_2_, and so on) and in a vacuum are often necessary. Gas-phase or surface reactions may be important. For example, owing to the dissociation of NH_3_ to H_2_ and inert N_2_ at elevated temperatures, processing conditions, such as an ammonia flow rate need to be carefully chosen. **b** Topochemical reactions to allow a rational design of structures (Fig. 1f). Low-temperature treatment of oxides with some reagents cause different anions to insert or exchange while maintaining the structural features. Multistep reactions have been also accessible^13,14^. **c** Epitaxial thin film growths and solvothermal reactions as a bottom-up process. Chemical bonding from ions of a substrate lattice yield metastable phases^19^. Local geometry can be manipulated by applying tensile or compressive strain from the substrate^20,21^. Solvothermal reactions offer an opportunity to prepare compounds with well-defined local structures. High throughput screening is possible with the Teflon pouch approach. **d** High-pressure reactions. High pressure can prevent some reagents from dissociation or evaporation (upper)^22–26,41^, and also stabilize dense structures (lower)^27^. **e** Computational tools. In particular, the rapid advancement of computational methods provides unprecedented opportunities for predicting and understanding mixed-anion compounds. DFT = density functional theory, MC = Monte Carlo, ML = machine learning, AI = artificial intelligence

Conventional inorganic materials are mostly oxides, due to the fact that the Earth’s atmosphere contains mainly reactive oxygen (and inert nitrogen). Thus, metal oxides are conventionally synthesized by high-temperature solid-state reactions, sometime called ‘heat and beat’ (or ‘shake and bake’) processing. A major difficulty in preparing mixed-anion compounds in the same way lies in the differing volatilities of precursors (oxides, chlorides, hydrides, and so on), so simple heating of mixed starting reagents often ends up with single-anion compounds, though some can be prepared in air (e.g., LaCl_3_ + 0.5O_2_ → LaOCl + Cl_2_). For this reason, the preparation of mixed anion compounds often requires controlled atmospheres, such as in vacuum or under various flowing gases (Cl_2_, F_2_, NH_3_, CS_2_, and so on) (Fig. 3a) or exploits alternative synthesis methods, including soft-chemistry (Fig. 3b), solvothermal synthesis, or thin-film growth techniques (Fig. 3c) and high-pressure synthesis (Fig. 3d).

For example, a high-temperature ammonolysis reaction (under NH_3_ flow) is employed^2^, instead of inert N_2_, to obtain many oxynitride semiconductors, including AMO_2_N (A = Ba, Sr, Ca; M = Ta, Nb) with a high dielectric constant due to the larger polarizability of nitrogen (Fig. 1e)^3^. However, the ammonolysis reaction involves the dissociation of NH_3_–N_2_ and H_2_ (Fig. 3a) and thus provides a highly reducing atmosphere, which gives a certain constraint on available metals. To increase the reactivity of ammonia, a microwave oven is used to generate an ammonia plasma^2^.

The high reactivity of the anionic species, often gaseous in elemental form, can conversely be an advantage in tailoring anions in extended solids at low temperature. Topochemical insertion and exchange reactions (Fig. 3b), which provide metastable mixed-anion phases from precursors (typically oxides) in a rational, chemically designed manner, have been developed over the last two decades^4^. A proper choice of reagents and host structures is essential in directing reactions in a desired way. Consider for example oxyfluorides: a F_2_ treatment can give an oxidative fluorination involving F-intercalation (e.g., LaSrMn^3+^O_4_ → LaSrMn^5+^O_4_F_2_), while poly(tetrafluoroethylene), known as Telfon, acts as a reductant and may lead to reductive fluorination involving O/F-exchange (e.g., RbLaNb^5+^_2_O_7_ → RbLaNb^4.5+^_2_O_6_F)^5,6^.

The hydride anion is strongly reductive in nature, with a large standard redox potential of −2.2 V (H^−^/H_2_ vs. SHE), so a transition metal oxyhydride appears impossible to stabilize. However, topochemical reaction using metal hydrides, such as CaH_2_ has opened a new avenue, yielding as the first example LaSrCoO_3_H_0.7_ (Co^1.7+^, *d*^7.3^) in 2002^7^. Density functional theory (DFT) calculations revealed the presence of fairly strong σ bonding between Co *e*_g_ and H 1*s* orbitals^8^. On the other hand, the formation of BaTiO_2.4_H_0.6_ (Ti^3.4+^; *d*^0.6^), SrVO_2_H (V^3+^; *d*^2^), and SrCrO_2_H (Cr^3+^; *d*^3^) is not readily rationalized since Ti/V/Cr *t*_2g_ and H 1*s* orbitals are orthogonal (Fig. 2c)^9–11^. Since all the known transition-metal oxyhydrides exist with alkali and alkaline earth elements^12^, inclusion of any highly electropositive cation appears to be needed to make hydrogen with its moderate electronegativity (Fig. 2b) become negatively charged. This may explain why TiO_2_ does not incorporate hydride.

The observation of H/D exchange in BaTiO_2.4_H_0.6_ when heated in deuterium gas at ~400 °C indicates the labile nature of H^–^ (Fig. 1f)^10^. The lability of hydride in BaTiO_2.4_H_0.6_ (and other oxyhydrides) enables further topochemical anion exchange reactions (Fig. 3b)^11,13,14^. When BaTiO_2.4_H_0.6_ is used as a precursor, the ammonolysis reaction temperature (>1000 °C) is remarkably lowered to 350 °C, yielding BaTiO_2.4_N_0.4_^13^. Even N_2_ flow at 400 °C gave the same product, demonstrating the ability of H^–^ to activate the nitrogen molecule. This hydride exchange chemistry is general, yielding other mixed-anion compounds, such as oxide-hydride-hydroxide BaTiO_2.5_H_0.25_(OH)_0.25_^14^.

Solvothermal synthesis is a synthetic method in which reactions occur in solution (i.e., water in the case of hydrothermal synthesis) inside a sealed vessel at temperatures near the boiling point of the solvent and pressures greater than atmospheric pressure^15^. Liquid-phase transport of the reactants allows for rapid nucleation and subsequent growth of a crystalline product with controlled morphology. This method produces crystals at lower temperatures and on shorter timescales than typical solid-state reactions. It also increases the likelihood of formation of mixed-anion compounds (e.g., halide hydroxides, oxyhalides), which are often unfavored at higher temperatures. Solvothermal syntheses have been very successful in producing materials with acentric coordination environments that lead to noncentrosymmetric (NCS) structures having desirable properties, such as piezoelectricity, pyroelectricity, and nonlinear optical activity^16^.

Direct fluorination of oxides with F_2_(g) or HF(g) is quite effective with minimal risk of side products. The handling of caustic, reactive gases, however, requires particularly specialized gas-phase reactors. In contrast, hydrothermal synthesis in hydrofluoric acid, or solutions of alkali fluorides, may be the easiest and safest route. The Teflon pouch approach is an efficient process to allow for fast development of discovery–based syntheses of new materials because various reactions can be performed in separate, small Teflon reaction pouches under identical, autogeneous conditions in an autoclave (Fig. 3c). Up to six reactions can be run in a 125 mL vessel.

Crystallographic long range ordering of oxide and fluoride anions has historically been a challenge, but materials based on anionic coordination polyhedra [MO_*m*_F_6–*m*_]^*n*–^ (where (*m*, *n*) = (1, 2) for M = V^5+^, Nb^5+^, Ta^5+^, (2, 2) for M = Mo^6+^, W^6+^, and (3, 3) for M = Mo^6+^)  have been solvothermally prepared without apparent anion-site disorder (Fig. 3c)^16^. In the ordered perovskite KNaNbOF_5_ and CsNaNbOF_5_ (with the general formula AM'MX_6 _(M' = alkali metal, M =  2^nd^ order Jahn-Teller *d*^0^ metal)), the interactions of the [NbOF_5_]^2–^ anion with the combination of Na/K or Na/Cs differ significantly. The NCS structure (KNaNbOF_5_) maintains a larger primary electronic distortion of the [NbOF_5_]^2–^ anion along with a low coordination number of the K^+^ ion, consistent with the largest bond strain index. In contrast, the Cs^+^ ions of the centrosymmetric structure (CsNaNbOF_5_) can exhibit higher coordination numbers and the [NbOF_5_]^2–^ anion exhibits a greatly reduced primary distortion. Theoretically, the group-theoretical method was applied to investigate anion ordering in the cubic perovskite, establishing 261 ordered low-symmetry structures, each with a unique space-group symmetry^17^. These idealized structures are considered as aristotypes with different derivatives formed by tilting of MO_6_ octahedra, providing a guide for designing NCS properties.

Thin film growth of oxides using pulsed laser deposition (PLD) or molecular beam epitaxy (MBE) is another useful bottom-up approach to construct desired artificial lattices, which has significantly contributed to the progress of condensed matter physics in the last two decades^18^. More rarely, thin film growth has been shown to be a promising method to prepare mixed-anion compounds, avoiding potential problems in anion diffusion. Oxynitrides films are fabricated by nitrogen plasma-assisted PLD, while polyvinylidene fluoride (PDVF) is used to topochemically convert oxide films to oxyfluoride ones. TaON films grown on a (LaAlO_3_)_0.3_(SrAl_0.5_Ta_0.5_O_3_)_0.7_ substrate adopt a metastable anatase structure with anion vacancies, leading to high-mobility electron transfer^19^. Tensile and compressive stresses from the substrate enables tailoring of the anion arrangement of a given structure. Compressively strained SrTaO_2_N films show a partial *cis*-to-*trans* conversion of TaO_4_N_2_ octahedra (Fig. 3c)^20^. An anion order/disorder transition can also be induced by strain engineering^21^. However, we note that there are still very few reports on mixed-anion films and most are thin film studies targeting optical (or surface) coating applications.

High pressure- and high-temperature conditions are typically used to stabilize dense materials through solid-state reactions or structural transformations. High-pressure reactions in sealed vessels prevent loss-of-volatile elements and so are particularly useful for anions, such as nitride to prevent loss-of-gaseous nitrogen (Fig. 3d). Autoclaves can be used for reactions under nitrogen up to kbar pressures, but many syntheses of oxynitrides have used direct reactions between solid oxides and nitrides (or oxynitrides) in multi-anvil presses where pressures can be extended to 10’s of kbar (GPa) values. The spinel Ga_3_O_3_N^22^ and AZrO_2_N perovskites (A = Pr, Nd, and Sm)^23^ were synthesized by direct solid-state reaction between oxides and nitrides or oxynitrides under GPa pressures. The use of solid reagents (instead of NH_3_) offers access to oxynitrides with middle-to-late transitions metals. A polar LiNbO_3_-type structure MnTaO_2_N with a helical spin order was recently synthesized at 6 GPa and 1400 °C^24^. A non-polar analog ZnTaO_2_N was also prepared^25^. New light atom materials have also been reported, such as the sphalerite-related boron oxynitride B_6_N_4_O_3_ synthesized from direct reaction between B_2_O_3_ and hexagonal-BN at 15 GPa and temperatures above 1900 °C^26^. Pressurization of baddeleyite-structured TaON drives a transition to a cotunnite-type structure with a very high-bulk modulus of 370 GPa (Fig. 3d)^27^.

## Chemical and structural analyses

Single crystal or powder diffraction methods are used to characterize many crystalline substances. A particular challenge for mixed-anion materials is to determine the distribution and degree of order-disorder of two or more anions. This complexity presents a challenge for both experiment and materials simulation (Fig. 3e), where equilibrium structures consisting of ordered or disordered anion configurations may be used for electronic structure calculations, e.g., those based on DFT or many-body methods. Ultimately to assess the properties of a mixed-anion material, the structure must be known. To that end, a number of structure-search algorithms, including cluster expansions^28^, special quasirandom structures^29^, and genetic algorithms^30^, frequently applied to multicomponent alloys and single-anion compounds, could be used to assess phase stability and solve structures in multi-anion compounds. In combination with experimental methods (below), a more complete description of the local and crystal structure can be obtained. These methods are also important for obtaining interaction energies for effective model Hamiltonians to describe ordering and ferroic transitions^31^.

Experimentally, the anion distribution may be studied directly using the scattering contrast between the anionic elements or indirectly through the different sizes or coordination environments of the anions in the structure. Direct X-ray scattering contrast is poor between elements from the same row of the periodic table, such as N/O/F or As/Se/Br, and neutron scattering may be useful in some cases, for example, to differentiate N and O which have respective neutron scattering lengths of 9.36 and 5.83 fm (Table 1) in oxynitrides. Neutron scattering also enables the positions of these light atoms to be determined more precisely in the presence of heavy metal atoms than is usually possible from X-ray refinements.

**Fig. 4:**
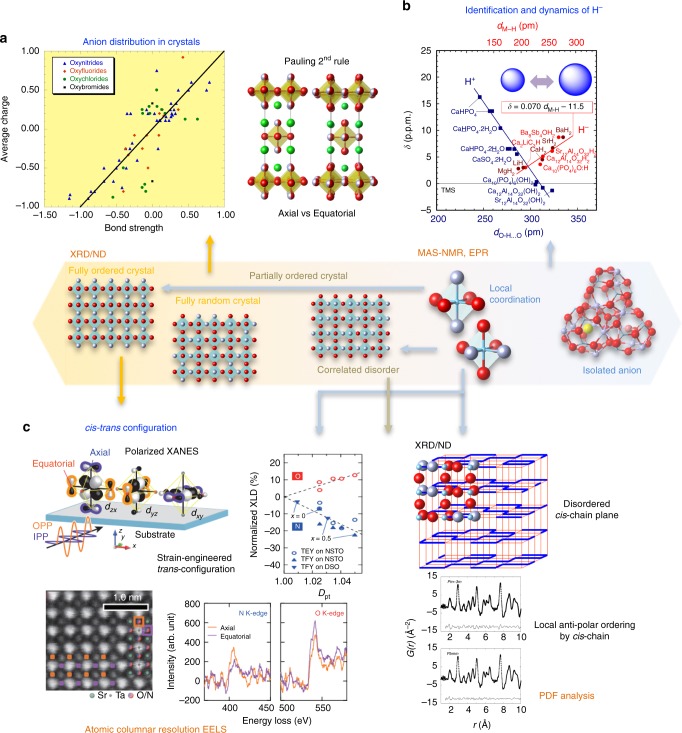
Chemical and structural characterizations for mixed-anion compounds. Hierarchical representations from long-range ordered structures to correlated disordered states, and to local structures. **a** Prediction of  anion distributions in mixed-anion (O, N, F, Cl, and Br) crystals based on the Pauling’s second rule: a correlation between the charge of an anion site with the calculated bond strength sums for the relevant site from X-ray diffraction (XRD) and neutron diffraction (ND) refinements^33^. For example, the apical site of the Nb(O,F)_6_ octahedron in K_2_NbO_3_F is favorably occupied by F^–^, while the equatorial site by N^3–^ in Sr_2_TaO_3_N. **b** Identification of H^–^ using the correlation between the chemical shift (δ) of ^1^H-NMR and the M–H distance (*d*_M–H_), where M is the neighboring cation (Fig. 2a)^44^. An opposite dependence is seen for OH^–^. **c** Characterization of *cis-* and *trans-*coordination in AMO_2_N perovskites (Fig. 1c). (Right) A tetragonal SrTaO_2_N structure (*P*4/*mmm*) with the equatorial site occupied equally by O/N and the apical site occupied completely by O, giving disordered *cis-*chains, where thick/thin lines correspond to M–N–M/M–O–M connections^34^. This model was deduced from the average site occupancies in **b**. The correlated anion disorder in AMON_2_ perovskites is chemically symmetric through reversal of O and N. PDF analysis of neutron total scattering data for BaTaO_2_N reveals local O/N ordering originated from favorable *cis*-configuration of TaO_4_N_2_ octahedra^39^. (Left) The *trans*-coordination in SrTaO_2_N film under lateral compressive strain is probed by polarized XANES and STEM-EELS^40^. Some data are reproduced with permission from each journal

Anions that have very similar X-ray and neutron scattering factors, such as oxide and fluoride may be distinguished by their structural environments if well-ordered within a crystal structure. Differences in formal charge and size are captured by the popular bond valence sum (BVS) method^32^, but even a simple approach based on apportioning ideal bond valences from Pauling’s second crystal rule was found to account for anion orders in many oxyhalides and oxynitrides (Fig. 4a)^33^. Increasing the formal anion charge tends to promote more covalent bonding to the metal cations and this can also enable anions to be distinguished; for example, vanadium forms very short ‘vanadyl’ bonds to oxide but not fluoride in V^4+^ and V^5+^ oxyfluorides.

Between the limits of fully ordered and randomly disordered anions, there are many cases of intermediate anion orders with local clustering or extended correlations that may give rise to non-random site occupancies in the averaged crystal structure. A particularly widespread example of such correlated disorder is found in AMO_2_N and AMON_2_ perovskite oxynitrides where layers of zig–zag MN chains (Fig. 4c) result from strong-covalent interactions between high-valence transition metals M and nitride anions that promote local *cis*-MN_2_ (or MO_4_N_2_) configurations (Fig. 1c, e). This order has been deduced from powder neutron refinements of O/N site occupancies in materials, such as SrMO_2_N (M = Nb, Ta)^34^, LaTaON_2_^35^, and AVO_2_N (A = Pr, Nd)^36,37^ perovskites. Local O/N correlations are also present in silicon oxynitrides where covalency tends to equalize the SiO_4–*n*_N_*n*_ compositions of all nitridosilicate tetrahedra, for example, in melilite-type Y_2_Si_3_O_3_N_4_^38^.

Analysis of total X-ray or neutron scattering data, including diffuse features from short-range correlations, as well as the Bragg scattering, has been used to construct the pair distribution function (PDF) of interatomic distances in many materials. Fitting of the PDF can be a powerful tool for revealing short range structural correlations in crystalline materials, as well as in amorphous substances^39^. Scattering or size contrast between anions can be used to determine their local order, for example, neutron PDF analysis revealed the prevalence of local *cis*-TaN_2_ configurations in the perovskite BaTaO_2_N (Fig. 4c)^40^.

Complementary information for analyzing the neutron- or X-ray-PDFs can be acquired by other techniques, such as electron energy loss spectroscopy (EELS) combined with scanning transmission electron microscopy (STEM), X-ray absorption near edge structure (XANES) of X-ray absorption spectroscopy (Fig. 4c), and magic angle-spinning (MAS) nuclear magnetic resonance (NMR) (Fig. 4b), which provide not only anion composition but also the local structures. As opposed to the above diffraction methods that may have difficulty in distinguishing among O, F, and N, state-of-the-art STEM-EELS can determine atomic occupancy with a resolution of each atomic column in a crystal lattice. This is particularly advantageous for thin film samples, in which crystal orientation is well controlled but precise structural analysis by diffraction methods is not as applicable. XANES is also effective for identifying the above elements and determining their chemical states. Perovskite (Ca_1–*x*_Sr_*x*_)TaO_2_N epitaxial films with controlled strains were analyzed using XANES with a polarized light source^41^. From the intensity of π-bonded states of O or N with Ta-5*d* via excitation from O and N core levels, it was concluded that N preferably takes the *trans* configuration in the TaO_4_N_2_ octahedron for compressive strain states, which was also supported by STEM-EELS and DFT calculations (Fig. 4c).

NMR has also been effective for (local) structural determination of mixed-anion compounds^42^. Structural determination of industrially important Si–Al–O–N materials (SiAlON), which are solid solutions between Si_3_N_4_ and Al_2_O_3_, by X-ray diffractometry is insufficient because X-ray scattering factors within the Si–Al and O–N pairs are similar; however, the high-resolution MAS-NMR method overcomes this challenge. Local coordination around the ^29^Si and ^27^Al nuclei was determined by MAS-NMR and their integration gives a full structural model for such oxynitride materials^43^ and, coupled with ab initio calculations, preferential Al–O clustering^44^.

High sensitivity is a hallmark of ^1^H-NMR, enabling detection of H^–^ with a concentration as low as 0.1% of the total anions. Coexistence of H^+^ (or OH^–^) and H^–^ ions in a single material is not trivial because their thermodynamic stability is different and depends on oxygen partial pressure, *p*(O_2_). However, these two species sometimes coexist due to non-equilibrium^14^ or high-temperature equilibrium^45^. Recent ^1^H-NMR has identified a ‘hidden’ hydride anion and its local environment in hydroxyl-oxides like apatite Ca_10_(PO_4_)_6_(OH)_2_^45^. Here, the size flexibility of H^–^ (Fig. 2a) substantially changes the electron density (and relevant magnetic field shielding) at ^1^H nuclei and hence the isotropic chemical shift of ^1^H-NMR (Fig. 4b).

Cage structures can incorporate various anionic species. Mayenite 12CaO·7Al_2_O_3_ with a positively charged cage structure is shown to host many mono- or divalent guest anions (F^–^, Cl^–^, S^2–^, O^–^, O_2_^–,^ O_2_^2–^, C_2_^2–^, NH^2–^, CN^–^, O^2–^, OH^–^, and H^–^) (Fig. 1h)^46^. Raman and electron paramagnetic spin resonance (EPR) measurements show that active oxygen species of O^–^, O_2_^–^, and O_2_^2–^, less stable than O^2–^ in oxide crystals and usually formed on surfaces transiently^47^, can stably exist in the cage. In a lightly hydride-doped mayenite, an irradiation of UV light induces a chemical reaction in the cage: H^–^ + O^2–^ ⇔ 2e^–^ + OH^–^ (Fig. 2d). Here, the e^–^ is confined within the cage, like *F*^+^ centers in alkali halides, and is responsible for a ‘permanent’ electrical conductivity as the reverse of the above reaction proceeds with a timescale of 10,000 years at room temperature^48^. Formation of transient atomic hydrogen during the photo-dissociation of H^–^ is monitored by EPR, revealing that its lifetime of the atomic hydrogen is a few minutes at 40 K^49^.

## Chemical properties

### Optical applications

Many oxides have a wide band gap and so are transparent. Valence band engineering according to Fig. 1b is useful to make them responsive to visible light, the main component of solar spectrum. When the oxide anion is substituted by other anions with less electronegativity like nitride (Table 1), the non-oxide *p* orbitals having high-potential energy extend the valence band and allow for visible-light absorption. Solid solutions of CaTaO_2_N and LaTaON_2_ perovskites have tuneable colors that range from yellow to red via orange (500–600 nm in wavelengths), depending on the composition of the solid solutions^50^. These oxynitrides are potential non-toxic alternatives to chalcogenide-based inorganic pigments.

**Fig. 5:**
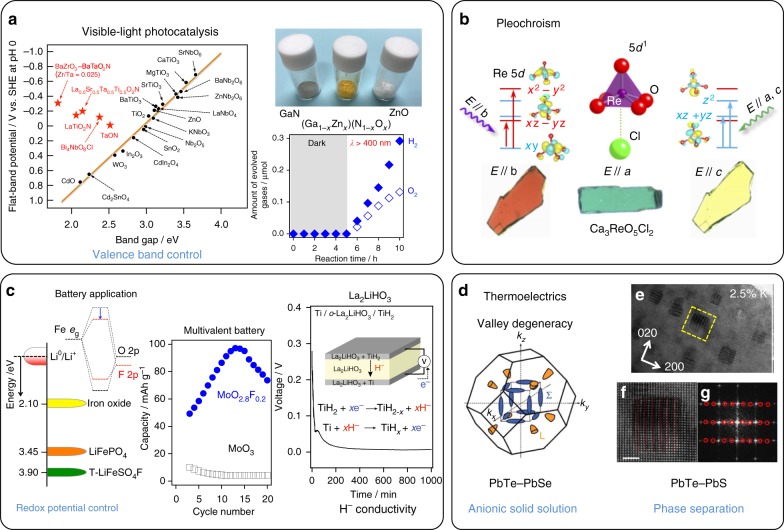
Mixed-anion driven chemical functions. **a** Visible-light photocatalysis (Fig. 1b). (Left) Flat-band potential as a function of the band gap, showing an empirical relation, *E*_FB_(NHE) ≈ 2.94—*E*_g_, for *d*^0^ or *d*^10^ oxide semiconductors (‘Scaife plot’).^51^ (Right) Powders of GaN, ZnO and their solid solution (Ga_0.58_Zn_0.42_)(N_0.58_O_0.42_), and a time course data for overall water splitting under visible light using (Ga_0.87_Zn_0.13_)(N_0.83_O_0.16_) with RuO_2_ nanoparticle cocatalyst^59^. **b** Pleochroism (Fig. 1a). Ca_3_ReO_5_Cl_2_ crystals showing different optical densities for incident light polarized along the *a*, *b*, and *c* axes^68^. **c** Battery applications. (Top left) Energy of the redox couples of iron phosphate frameworks relative to the Fermi level of metallic lithium (Fig. 1a, b)^72^. (Bottom left) Capacity versus cycle number for MoO_2.8_F_0.2_ over the first 18 cycles (Fig. 1b, e)^76^. (Right) A pure H^–^ conductivity^71^. Discharge curve for a solid-state battery with the Ti/La_2_LiHO_3_/TiH_2_ structure (Fig. 2a, b). **d** Thermoelectrics. (Left) Brillouin zone of PbTe_1–*x*_Se_*x*_, where the anion tuning allows creation of low-degeneracy hole pockets (orange) and the high-degeneracy hole pockets (blue)^81^. (Right) Microstructures for nanoscale precipitates of a phase-segregated (2.5% K-doped) PbTe_0.7_S_0.3_^80^. The lower panels show an enlarged image of cubic precipitates with the three-layered structure and its Fourier-transformed image. Some data shown here are reproduced with permission from each journal

This strategy may be of particular importance for finding a photocatalyst which can split water to produce H_2_ and O_2_ under visible light. Otherwise, if oxides with a small band gap of < 3 eV (corresponding to *λ* > 400 nm) are used, the conduction band minimum (CBM, or flat-band potential) becomes more positive than the water reduction potential (0 V vs. NHE (normal hydrogen electrode) at pH 0), a limitation shown by Scaife^50^ (Fig. 5a). So far, various oxynitrides and oxysulfides that overcome this limitation have shown water splitting performance^52–54^. Some of them (e.g., ZrO_2_-grafted TaON) were found to be a useful component for Z-scheme type water splitting^55^ and CO_2_ reduction with the aid of a functional metal complex^56^.

Unexpected changes in electronic structure are often found in mixed-anion compounds, which presents a challenge to predictive materials theory. Methods based on DFT require appropriate exchange-correlation functionals^57,58^ to accurately describe the mixed bonding character presented in these materials. Alloying wide-gap semiconductors, GaN and ZnO, results in an unprecedented yellowish powder (Fig. 5a), and this provides the first reproducible example of visible-light-driven overall water splitting^59^. Loaded with nanoparticulate Rh_2_O_3_–Cr_2_O_3_ that works as an active site for H_2_ evolution, (Ga_1–*x*_Zn_*x*_)(N_1–*x*_O_*x*_) exhibited H_2_ and O_2_ evolution for >3 months^60^. One of the drawbacks of mixed anion photocatalysts in general is their instability against photo-induced holes. This is seen even in (Ga_1–*x*_Zn_*x*_)(N_1–*x*_O_*x*_), where the photo-induced holes oxidize the N^3–^ anion, degrading its photocatalytic activity by self-decomposition^60^. Bi_4_NbO_8_Cl, a Sillen–Aurivillius layered perovskite, was recently shown to stably oxidize water without any surface modifications. The observed stability is attributed to highly dispersive O-2*p* orbitals (dominating the VBM instead of Cl-3*p*)^61^. A recent study on a series of layered bismuth oxyhalides has revealed that Madelung site potentials of anions capture essential features of the valence band structures of these materials, enabling prediction and design of the valence band structures by manipulating the stacking sequence of layers (Fig. 1g)^62^.

Oxynitrides doped with rare earth elements show photoluminescence. Here, substitution of O^2–^ for N^3–^ gives a greater CFS of the 5*d* levels of rare earth elements, such as Eu^2+^ (Fig. 1a), extending the excitation and emission peaks to longer wavelengths. SiAlON, (Si_3–*x*_Al_*x*_)(N_4–*x*_O_*x*_):Eu^2+^, and related phosphors undergo photoexcitation by absorbing blue light, and emitting yellow light, and hence are used in phosphor-converted white-light emitting LED lamps (WLEDs)^63^. Other important SiAlON-related phosphors used in WLEDs are the ASi_2_O_2_N_2_:Eu^2+^ and A_2_Si_5_N_8_:Eu^2+^ families (A = Ca, Sr, and Ba)^64^, the latter can be oxide-doped with Al^3+^ providing charge compensation in A_2_Si_5−*x*_Al_*x*_N_8−*x*_O_*x*_:Eu^2+^ (*x* = 0–1)^65^. The high thermal and chemical stability arising from covalent M−N bonding (Fig. 1e) leads to practical applications. Similar chemical tuning has been applied for oxyfluoride type solid solutions, such as A^II^_3–*x*_A^III^_*x*_MO_4_F family with A = Sr, Ca, Ba and M = Al, Ga (e.g., (Sr,Ba)_2.975_Ce_0.025_AlO_4_F) ^66,67^.

Another interesting feature from the mixed-anion system is pleochroism, recently found in Ca_3_ReO_5_Cl_2_ with the Re^6+^ ion in a 5*d*^1^ configuration (Fig. 5b)^68^. The heavily distorted octahedral coordination of Re^6+^ by one Cl^–^ and five O^2–^ anions along with the spatially extended 5*d* orbitals gives rise to unique CFS energy levels (Fig. 1a), much greater than for 3*d* orbitals owing to stronger electrostatic interactions exerted from the ligands. The uni-directional alignment of these octahedra along the *c*-axis makes the *d–d* transitions highly anisotropic. As a result, this compound exhibits very different colors depending on the viewing direction, i.e., distinct pleochroism.

### Anion conductors

Certain anions are mobile in solids. The merit of a mixed-anion material is that it allows for anion diffusion by one (more ionic, less highly charged) anion and structural stability by the other (more covalent, more highly charged) anion (Fig. 1e, f). This concept can be directly assessed using electronic structure methods, where calculations of intrinsic defect levels and diffusion barriers^69^ can be correlated with changes in the anion lattice. A layered lanthanum oxychloride LaOCl is a Cl-ion conductor^70^. While La_2_O_3_ and LaCl_3_ are both sensitive to moisture, a critical disadvantage for practical applications, LaOCl is water-insoluble and exhibits Cl conductivity. An aliovalent Ca–for–La substitution generates vacancies at the chloride site and hence the Cl^–^ conductivity is improved.

H^–^ anion conductors are expected to provide high-energy storage and conversion devices because H^–^ has an appropriate ionic size for fast diffusion (Fig. 2a), a low electronegativity (Fig. 2b) and a high-standard redox potential of H^–^/H_2_ (−2.3 V), close to that of Mg/Mg^2+^ (−2.4 V). A pure H^–^ conduction in K_2_NiF_4_-type La_2_LiHO_3_ has recently been demonstrated, using an all-solid-state TiH_2_/La_2_LiHO_3_/Ti cell (Fig. 5c)^71^. The two-dimensional (2D) H^–^ diffusion is further facilitated by introducing H^–^ vacancies, leading to the activation energy of 68.4 kJ mol^−1^ for La_0.6_Sr_1.4_LiH_1.6_O_2_.

### Battery electrodes

Mixed-anion chemistry of oxyfluorides offers a new handle to tune the redox potential of battery electrodes. Here, instead of ‘direct’ valence (anion) band control described in Fig. 1b, anion substitution enables an ‘indirect’ manipulation of the cation band. The redox potential of the LiFeSO_4_F phase (tavorite) is higher than the LiFePO_4_ phase (olivine) by 750 mV^72^. This primarily results from the weaker (more ionic) Fe–F bond as compared with the Fe–O bond (Fig. 1e), which stabilizes the anti-bonding band of Fe *e*_g_ orbitals (Fig. 5c). Furthermore, Ag_2_V_2_O_6_F_2_ (SVOF) is a battery material potentially used in cardiac defibrillators owing to a fast discharge rate and high-current density^73^. The silver density in SVOF is greater than that of the currently used industry standard cathode material Ag_2_V_4_O_11_ (SVO)^74^ and thus the current density above 3 V for SVOF (148 mAh/g) is greater than that for SVO (100 mAh/g). The current density above 3 V is sufficient and the potential at which it is delivered (3.52 V) is 300 mV greater than SVO owing to the fluoride incorporation (Fig. 1b).

Multivalent batteries exhibit a number of potentially valuable advantages compared to current lithium technology. The first functional multivalent battery was constructed in 2000; this prototype used a magnesium metal anode against a low-voltage Chevrel phase cathode^75^. A significant barrier to the adoption of magnesium batteries is the lack of an available high-voltage cathode that can reversibly intercalate magnesium. Cathodes composed of layered molybdenum fluoro-bronze are found to reversibly intercalate magnesium^76^. MoO_2.8_F_0.2_, combined with a Mg-based electrolyte, gave a reversible capacity of nearly 80 mAh/g, an order of magnitude higher than isostructural α-MoO_3_ with a similar particle size (Fig. 5c). First-principles calculations revealed that the incorporation of fluoride within the crystal lattice reduces nearby molybdenum ions, enhancing in-plane electronic conductivity^77^. The associated increase in electronic screening reduces the activation barrier for Mg ion diffusion but yet does not significantly lower the voltage.

### Thermoelectric materials

Thermoelectric materials enable direct conversion between thermal and electrical energy. Optimal materials with a high figure of merit ZT have a high Seebeck coefficient and electronic conductivity in combination with a low-thermal conductivity. BiCuSeO with (Cu_2_Se_2_)^2−^ layers alternately stacked with (Bi_2_O_2_)^2+^ layers (Fig. 1g), is a promising thermoelectric material, where one layer is responsible for electric conduction, while another lowers thermal conductivity^78^.

Nanostructuring which may be based on local segregation of anions is another effective means to reduce phonon thermal conductivity. The PbTe–PbS system exhibits phase separation (spinodal decomposition), owing to a large difference in the anion sizes (Hume-Rothery rules)^79^. The resultant PbTe-rich and PbS-rich regions form dissimilar nanostructures with interphase boundaries that act as effective scattering centers for short-wavelength phonons (Fig. 5d). A nominal composition of PbTe_0.7_S_0.3_ doped with 2.5% K achieved a figure-of-merit ZT of > 2 over a wide temperature range from 400 to 650 °C^80^. On contrary, a complete solid solution is formed in the PbTe–PbSe system. By tuning the anionic composition in Pb(Te_1–*x*_Se_*x*_), the electronic band structure exhibits high-valley degeneracy (Fig. 5d), leading to an optimized ZT value of 1.8 at 577 °C^81^.

## Physical properties

**Fig. 6:**
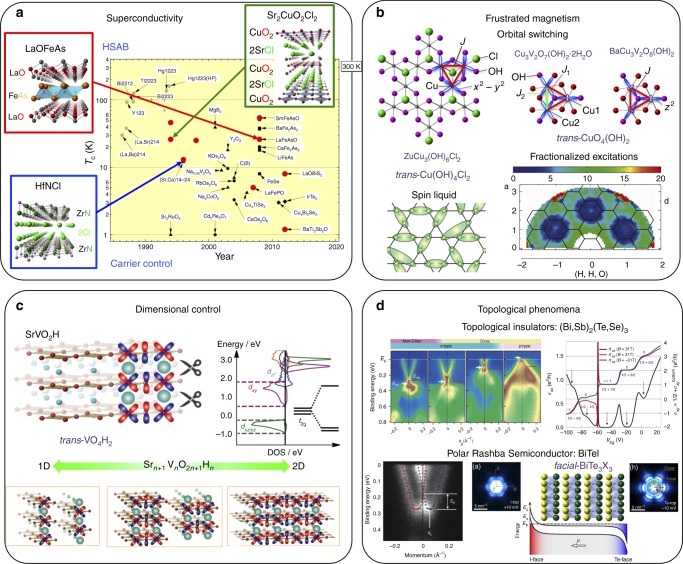
Mixed-anion driven physical functions. **a** Superconducting transition temperatures as a function of the year of discovery, where symbols of mixed-anion compounds are highlighted in color. Layered structures of parent high-*T*_c_ superconductors HfNCl^83^, LaOFeAs^104^ and Sr_2_CuO_2_Cl_2_^89^ are shown (Fig. 1g). **b** (Top) Geometrical frustration in ZnCu_3_(OH)_6_Cl_2_, Cu_3_V_2_O_7_(OH)_2_·2H_2_O and BaCu_3_V_2_O_8_(OH)_2_ with the *S* = 1/2 kagomé lattice^85,87^. A Cu-triangle unit is formed by the chlorine anion of three *trans*-Cu(OH)_4_Cl_2_ octahedra in the former, while sharing the OH anion of three *trans*-CuO_4_(OH)_2_ octahedra in the latter two compounds. Different orbital-ordering patterns appear in these compounds, leading to various exotic quantum states. (Bottom) A spin liquid ground state and inelastic neutron scattering on ZnCu_3_(OH)_6_Cl_2_ showing fractionalized excitations^86^. **c** (Upper) Crystal and electronic structures of SrV^III^O_2_H with *trans*-VO_4_H_2_ octahedra^9^. H^–^ 1*s* orbitals, orthogonal with V *t*_2g_ orbitals act as orbital scissors (or π-blockers), resulting in 2D electronic structures (Fig. 2c). (Lower) 2D-to-1D crossover in serial *n*-legged spin ladders, Sr_*n*+1_V_*n*_O_2*n*+1_H_*n*_ (Fig. 1g)^90^. **d** (Upper left) Band dispersions of the cation/anion co-substituted (Bi,Sb)_2_(Te,Se)_3_ with a tunable Dirac cone^91^. (Upper right) Topological surface state quantum Hall effect in the intrinsic topological insulator (Bi,Sb)_2_(Te,Se)_3_^92^. (Lower left) Giant bulk Rashba effect in BiTeI with polar *facial*-BiTe_3_I_3_ octahedral layers (Fig. 1d)^93^. (Lower right) Spectroscopic imaging scanning tunneling microscopy of BiTeI evidencing the ambipolar 2D carriers at the surface, indicating the formation of lateral *p*–*n* junctions^94^. Some data shown here are reproduced with permission from each journal

Ordering of two anions within a material often leads to low dimensionality in structural and physical properties. Layering of different anion types (Fig. 1g) is common and leads to 2D conductivity or magnetic correlations when cations with unpaired electrons are present. The ZrCuSiAs structure type is a flexible arrangement that allows two different anions and cations to segregate into distinct layers according to HSAB (hard and soft acids and bases) principles. Many mixed-anion materials adopt the ZrCuSiAs type, notably the LnFeAsO family of layered magnetic conductors and (when suitably doped) high-*T*_c_ superconductors (Fig. 6a), the p-type semiconductor LaCuSO, the ferromagnetic Kondo material CeRuPO, and the Ag-ion conductor LaAgSO^82^. Layered order of nitride and halide anions in MNX materials (M = Ti, Zr, and Hf; X = Cl, Br, and I) results in X-M-N-N-M-X slabs separated by van der Waals gaps (Fig. 6a) into which cations such as lithium are intercalated, leading to conductivity and superconductivity^83^.

Multiple anions and their ratios may be used to control dimensionality and connectivity of magnetic interactions. V^4+^ and Cu^2+^ both have spin *S* = 1/2 and so are of interest for quantum magnetic and superconducting properties, especially in low-dimensional structures that are often found in mixed-anion materials. In the early copper oxide superconductor studies, two copper oxyhalides, Sr_2_CuO_2_F_2+δ_^84^ and (Ca,Na)_2_CuO_2_Cl_2_^85^, played a role in understanding the superconducting mechanism (Fig. 6a). Although, these compounds possess F^–^ and Cl^–^ ions instead of O^2–^ ions at the apical site above and below the Cu^2+^ ions, they are superconducting with *T*_c_ = 46 and 26 K, respectively. This fact challenged the theoretical models proposing a vital role of the apical oxygen in the superconducting mechanism. Now it is well established that the high-*T*_c_ superconductivity occurs within the CuO_2_ sheet having a strong covalency between the Cu $d_{x^2 - y^2}$ and O 2*p*_σ_ states, while the apical-site anions (oxide ions) are more ionic (Fig. 1e), resulting in the 2D electronic state. In V^4+^ oxyfluorides, the V = O vanadyl oxide anions do not link to other cations whereas fluorides readily form V–F–V bridges, enabling many structural topologies to be achieved. DQVOF (Diammonium Quinuclidinium Vanadium OxyFluoride; [NH_4_]_2_[C_7_H_14_N][V_7_O_6_F_18_]) is notable as a geometrically frustrated kagomé bilayer material with a gapless spin liquid ground state, instead of the conventional Néel order (Fig. 6b)^86^. Various synthetic copper minerals with Cu^2+^ (*S* = 1/2 ion) and mixed anions have been studied as geometrically frustrated quantum magnets that can also show exotic ground states. A good example is herbertsmithite, ZnCu_3_(OH)_6_Cl_2_ (Fig. 6b), in which the Cu^2+^ ion is coordinated by two axial Cl^–^ ions and four equatorial OH^–^ ions with its spin residing on the $d_{x^2 - y^2}$ orbital^87^. The Cu^2+^ spins form a 2D kagomé lattice and are coupled to each other by strong superexchange interactions only via the OH^–^ ions. The compound exhibits no long-range order down to 50 mK with fractionalized excitations (Fig. 6b)^88^, owing to the strong frustration on the kagomé lattice. Volborthite Cu_3_V_2_O_7_(OH)_2_·2H_2_O and vesignieite BaCu_3_V_2_O_8_(OH)_2_ with *trans*-CuO_4_(OH)_2_ octahedra having different orbital arrangements composed of $d_{x^2 - y^2}$/$d_{z^2}$ and $d_{z^2}$ orbitals, respectively, enrich the phase diagram of the kagomé antiferromagnet^89^.

The lack of *p* orbitals in the valence shell of H^–^ (1*s*) effectively blocks the π-symmetry exchange pathways (Fig. 2c), a situation occurring in SrV^III^O_2_H with (*t*_2g_)^2^, where the in-plane exchange via V_dπ_–O_pπ_–V_dπ_ is much greater than the out-of-plane one via V_dπ_–H_1s_–V_dπ_ interactions (Fig. 6c)^9^. The application of pressure to the Mott insulator drives a transition to a metal at ~50 GPa. Interestingly, despite the enormous compressibility of hydride (Fig. 2a), which is twice as compressible as oxide (Fig. 2a), the electronic structure of the metallic phase is quasi-2D, meaning that the hydride ligand acts as a ‘π-blocker’. The dimensional control from 2D to 1D is possible in the *n*-legged spin ladder oxyhydrides Sr_*n*+1_V_*n*_O_2*n*+1_H_*n*_ (*n* = 1, 2,.., ∞) (Fig. 1g)^90^.

During the last decade, there has been remarkable progress in physics involving topological phases of matter, for which mixed-anion compounds play crucial roles in advancing this field. Binary chalcogenides Bi_2_Se_3_ and Bi_2_Te_3_ were thought to be potential three-dimensional topological insulators, but both suffered from native point defects and unintentional carrier doping. Alloying with these two compounds along with Sb-for-Bi substitution has established a highly insulating bulk and accessible Dirac carriers, accompanied by the observation of a sign change of the Dirac carriers (holes vs electrons) with chemical potential (Fig. 6d)^91^. The precise carrier control has been also utilized to achieve a topological surface state quantum Hall effect (Fig. 6d)^92^.

The layered polar semiconductor BiTeI shows a huge bulk Rashba-type spin splitting (Fig. 6d) that arises from the strong inversion asymmetry along the trigonal *c*-axis induced by distinct covalent Bi-Te and ionic Bi-I bonds in the *facial*-BiTe_3_I_3_ coordination (Fig. 1d)^93^. This built-in bulk polarity induces 2D electronic surface structures with heavy depleted (I-termination) and accumulated (Te-termination) electrons forming *p*–*n* junctions (Fig. 6d)^94^. Although BiTeI is a nontopological insulator at ambient pressure, it is proposed that the strong spin-orbit interaction allows a pressure-induced transition to a strong topological insulator, where, due to the broken inversion symmetry, a Weyl semimetal emerges between the two insulating phases^95^.

## Outlook

Increasing interest in solids based on mixed anions is expected to lead to new materials, some of which will make significant contributions to catalysis, energy conversion, and electronic devices, and will ultimately benefit industry in the coming decades. Functionality based on the earth-abundant, light elements usually present as anionic species (O, N, H, S, Cl, and so on) also offers the advantage of avoiding the inherent scarcity problems of metals, such as lanthanides. The metastability of mixed-anion compounds increases the complexity of synthesis and can limit the ways in which these materials can be used in devices. Therefore, chemically stabilizing these phases has to be considered when they are adapted for applications.

Synthetically, there will still be much room to develop methodologies. For example, multiple synthetic tools are used together (e.g., topochemical reaction under high pressure) or in a multistep process (e.g., solvothermal reaction followed by electrochemical reaction), both providing further platforms to manipulate multiple anions in extended solids. One of the important challenges is how to control anion order/disorder—one idea may be to utilize the size flexibility of hydride (Fig. 2a) to induce an order-disorder transition by (chemical) pressure. Furthermore, exploratory synthesis can be joined with computational tools ranging from DFT calculations to machine learning to expedite the screening process.

Regarding catalysis, this review has focused on visible-light-driven water splitting, but we believe that mixed-anion compounds can offer a variety of new possibilities, which would provide a large impact on chemical industry. In fact, an oxyhydride BaTiO_2.5_H_0.5_ has been very recently found to be an active catalyst for ammonia synthesis, which is remarkable given that Ti has been regarded as a ‘dead’ element in terms of heterogeneous catalysis^96^. The lability of hydride (Fig. 1f) may be responsible for this catalytic activity. Introduction of a new anion, not limited to hydride, into oxides will therefore be a useful strategy to explore a new catalytic function of ‘inert’ oxides. In situ and in operando analytic techniques will benefit and improve our understanding of these functions arising from mixed anion materials. The integration of DFT and machine learning and experiment can lead to the most likely reaction mechanism, and also provide new concepts or guiding principles to be added in Figs. 1 and 2.

Most functional mixed-anion materials known to date, and providing the focus of this review, are oxide based, although non-oxide mixed-anion systems may also provide novel phases and phenomena^83,97–99^. The additional inclusion of molecular anions (e.g., O_2_^–^, BH_4_^–^) can give rise to new aspects of anion-based materials (Fig. 1h)^100–102^. For instance, the use of anisotropic anions, such as O_2_^2–^ or S_2_^2–^ will result in local symmetry breaking and alter the hybridization with coordinating cations. Furthermore, mixed anions in surface, 2D-sheet materials^99^, interfaces, porous and nano materials, and amorphous systems are an important area for both fundamental and applied research.

There is still much to discover about the scientific principles and technological applications of mixed-anion materials. It means that the future prospects of mixed-anion materials are largely unknown at this time and this is what precisely makes the field so interesting moving forward. P. W. Anderson famously proposed that ‘More is Different’; in the world of anion-based materials we analogously conclude that ‘Mixed is Different’.

### Box 1. From oxides to mixed-anion compounds

Applications of oxides date back to prehistoric times, when our ancestors found useful properties from natural stones including, e.g., arrowheads, magnets, pigments, gems, and even medicines. Subsequent efforts have been devoted to improvements and hunting for new functions. The 20th century was a prosperous era, with discoveries of synthetic oxides that sustain modern technology, as exemplified by the ferroelectric BaTiO_3_, yttria-stabilized zirconia (YSZ) for solid oxide fuel cells, and Li_*x*_MnO_2_, a cathode material for lithium batteries. The successful story of oxides (and other single-anion compounds, such as fluorides, nitrides, and chlorides) is largely due to their stability and ease of synthesis, along with development of structural characterization techniques, such as X-ray diffraction. Numerous inorganic compounds (51,856 oxides, 1581 nitrides, 2978 fluorides in the Inorganic Crystal Structure Database (ICSD, https://icsd.fiz-karlsruhe.de), as of 5 October 2017) have been reported, most of which can be prepared by high-temperature solid-state reactions over 1000 °C. A result of extensive research over the last century is that new materials accessible by ‘heat and beat’ exploration of new cation combinations may be exhausted soon.

Focusing on the anions within a compound offers a solution to this problem. This can enhance the possible combinations of elements, but also offers more diversity. Cation-based compounds are based on common coordination polyhedra as building units (e.g., CuO_4_ square planes). However, if several oxide anions are replaced with other anions, new and unusual coordination geometries may result. When these polyhedra, as new building blocks, are arranged to form an extended array, one can expect enhanced properties or fundamentally new phenomena. Since anions exhibit different characteristics (e.g., ionic radii, valence, polarizability, and electronegativity), selecting different anions can introduce a new dimension of flexibility for materials design and function. Despite such possibilities, the number of mixed-anion compounds available are limited: the number of recorded materials in ICSD are 1266 for oxyfluorides, 612 for oxynitrides, 47 for oxyhydrides, 655 oxychalcogenides, and 312 oxypnictides. Note that mixed-anion compounds do not necessarily possess a heteroleptic coordination geometry around a transition metal. For example, a number of structures are comprised of alternating layers, each with a homoleptic coordination by a different anion, as found in Sr_2_MnO_2_Cu_1.5_S_2_ with alternating Sr_2_MnO_2_ and Cu_1.5_S_2_ layers^102^.

Although some excellent overviews of mixed anion compounds have been provided^2,5,12,16,81,82,102^, each covers relatively narrow range of materials and disciplines. This review article is attempting to capture the broader fundamentals of these materials and draw new insights among materials classes.
